# Effect of Tea Seed Oil on In Vitro Rumen Fermentation, Nutrient Degradability, and Microbial Profile in Water Buffalo

**DOI:** 10.3390/microorganisms11081981

**Published:** 2023-08-01

**Authors:** Huade Xie, Fanquan Zeng, Yanxia Guo, Lijuan Peng, Xianqing Luo, Chengjian Yang

**Affiliations:** Guangxi Key Laboratory of Buffalo Genetics, Reproduction and Breeding, Guangxi Buffalo Research Institute, Nanning 530001, China; xiehuade093@163.com (H.X.); 13471010613@163.com (F.Z.); gyxlq0417@163.com (Y.G.); lijuanpeng2000@163.com (L.P.); 19101477179@163.com (X.L.)

**Keywords:** tea seed oil, in vitro rumen fermentation, nutrients degradability, methane, microbial profile

## Abstract

Tea seed oil (TSO) was investigated for its effects on rumen fermentation and in vitro parameters of bacterial communities in water buffalo diets containing *Siraitia grosvenorii* and soybean residues. TSO was added at rates of 0% (control group (CT)), 0.5% (T1), 1% (T2), and 2% (T3) of the in vitro fermentation substrate weight (dry matter (DM) basis). T2 and T3 had significantly lower acetate and total volatile fatty acid contents but a significantly higher microbial crude protein content than CT. The lowest NH_3_-N content was observed in T1 and T2. Treatment significantly increased DM digestibility, with the highest percentage observed in T2. T2 showed significantly higher crude protein digestibility than CT. TSO supplementation significantly increased the C18:2n6c, C18:2 trans-10, cis-12, and C20:4n6 concentrations compared to those in CT. The total number of bacteria was significantly lower in T2 than in CT. TSO supplementation decreased the total bacteria, fungi, and methanogen populations but increased rumen microorganism diversity and richness. In conclusion, TSO can regulate the number and flora of rumen microorganisms through antimicrobial activity, thereby affecting rumen fermentation patterns, reducing methane production, and improving nutrient digestibility, and an optimal supplementation rate appears to be achieved with 1% TSO (DM basis).

## 1. Introduction

*Siraitia grosvenorii* (Luo hanguo or monk fruit, SG) is a valuable and traditional medicinal herb grown in China for more than two centuries and occurs mainly in the Guangxi province of China. SG contains rich functional and nutritional chemical components such as glycosides, flavonoids, polysaccharides, proteins, and essential oils [1]. SG residue (SGR) is a by-product of extracting SG glycosides and still contains functional components and protein, polysaccharide, and antioxidant substances [2]. Today, when antibiotics are prohibited and non-resistant production is advocated, SGR is a safe and high-quality functional raw material and has certain application value in animal feeding [3].

Soybean residue (SR) is a by-product of the processing of tofu, soymilk, and other soy products using soybeans as raw materials [4]. With the high demand for tofu and soy products in China, the corresponding production of SR is high, producing approximately 70 million tons of SR every year [5,6]. SR contains nutrients such as protein, sugar, and fat, with a high development and utilization value [7,8,9]. Using SR as animal feed can reduce production costs and is a cost-effective protein supplement. Furthermore, feeding livestock and poultry with SR instead of some protein feed not only addresses resource waste but also reduces feed costs, achieving a virtuous ecological cycle of production of by-products, production, environmental protection, and comprehensive utilization, which will generate significant social benefits.

Ruminant livestock systems contribute substantially to the emissions of methane (CH_4_), a potent greenhouse gas; a portion of the ingested energy (2–15%) is emitted as CH_4_ by rumen fermentation [10]. Thus, reasonable regulation of rumen fermentation can not only reduce the waste from feed resources but also improve the production performance of ruminants to a certain extent. With the prohibition of growth-promoting antibiotics in animal feed, plant extracts have attracted widespread attention as a new type of “green” feed additive. Essential oils (EOs) have been proposed as substitutes for chemical feed additives due to their potential as rumen fermentation modifiers; their antibacterial, antifungal, and antioxidant properties make them useful as natural additives in animal feeds [11,12]. Furthermore, EOs may affect rumen development due to their effect on microorganism populations and may subsequently change rumen fermentation profiles, improving feed efficiency [13,14].

Tea seed oil (TSO) refers to the edible oil derived from the seeds of *Camellia oleifera.* TSO is rich in monounsaturated oleic acid, with concentrations ranging from 60 to 80%, and various bioactive compounds [15]. The seeds of plants usually contain various antioxidant factors; the naturally occurring active components in tea seeds are different from other kinds of oil seeds [16]. The potent antioxidant activity of TSO confers protection from free-radical-related diseases [17]. Thus, TSO can be considered a potential alternative for rumen fermentation modifiers and natural additives. Therefore, we hypothesized that feeding water buffalo SGR and SR together with EO additives could complement dietary nutrients and reduce CH_4_ production while improving feed digestibility and fermentation characteristics, thereby making it possible to effectively use locally available feed resources. However, to our knowledge, relatively little information is available on in vitro ruminal fermentation characteristics of SGR and SR mixtures with or without TSO.

Therefore, the study objective was to evaluate the effects of increasing TSO supplementation rates on water buffalo in vitro fermentation parameters, fatty acid, gas production, and ruminal bacterial communities under a diet containing SGR and SR.

## 2. Materials and Methods

### 2.1. Substrates and Treatments

The substrate was composed of 70% SGR and 30% SR on a dry matter (DM) basis. Details of the chemical composition of the substrate are given in Table 1. Four treatments containing 1 g of the substrate (70% SGR and 30% SR mixture) were supplemented with 0% (CT), 0.5% (T1), 1% (T2), and 2% (T3) TSO (>99% purity; Yihai Kerry Arawana Holdings Co, Ltd., Shanghai, China) and were applied in a completely randomized design (Table 2). Each treatment group had six incubation bottles as replicates per run.

### 2.2. In Vitro Batch Culture

Three healthy water buffalo (average body weight 650 ± 50 kg) fitted with permanent rumen cannulas were used as ruminal fluid donors. The buffalo were cared for according to the guidelines of the Ethics Committee of the Guangxi Buffalo Research Institute, China. Rumen fluid was collected from the buffalo before morning feeding and strained through four layers of cheesecloth and combined on an equal volume basis. The combined filtrate was mixed with CO_2_-bubbled artificial saliva at a 1:2 volume ratio. The artificial saliva was prepared anaerobically as described by Guo et al. [18]. A total of 50 mL of buffered ruminal fluid was transferred to serum bottles (180 mL) containing 1 g of ground sample with or without TSO and was flushed with CO_2_ to preserve an anaerobic environment. The bottles were capped with a butyl rubber stopper and sealed with an aluminum cap. Incubation was performed at 39 °C for 72 h in a water bath with a shaker (100 strokes/min). Two experimental runs were performed for two consecutive weeks using the same experimental conditions.

### 2.3. Total Gas, Hydrogen (H_2_), and CH_4_ Production Analysis

The gas production was measured from the serum bottles using air syringes as described by Guo et al. [18]. At the same time as gas measurement, the H_2_ and CH_4_ production was measured using gas chromatography (GC) (8860, Agilent Technologies Co., Ltd., Shanghai, China). The cumulative H_2_ and CH_4_ production in 72 h was the sum of the actual H_2_ and CH_4_ production of the incubation bottle at each time point.

### 2.4. Rumen Fermentation Parameter Analysis

After incubation for 72 h, fermentation was stopped by swirling the bottles in ice. The pH was measured immediately after opening the bottles using a pH meter (PH8180–0–00; Smart Sensor Co., Ltd., Dongguan, China). Separate subsamples of the supernatant were used to determine volatile fatty acid (VFA) fractions using GC (7890A; Agilent Technologies, Santa Clara, CA, USA) as described by Azizi et al. [19]. The microbial crude protein (MCP) content was analyzed by colorimetry using an ultraviolet-visible spectrophotometer (PE Lambda 35, Shanghai Pudi Biotechnology Co., Ltd., Shanghai, China). The ammonia N (NH_3_-N) was determined using the phenol-hypochlorite procedure [20].

### 2.5. Fatty Acid Analysis

The extraction, methylation, and determination of medium and long-chain fatty acids in rumen fluid using the GC system (Trace1300, Thermo Fisher Scientific, Waltham, MA, USA) were determined using a GC hydrogen flame ionization detector (GC–MS), and automatic sampler (ISQ7000, Thermo Fisher Scientific, Waltham, MA, USA) as described by Guo et al. [21]. The fatty acid methyl ester was determined using a capillary column, and the concentration of long-chain fatty acids was detected using C19:0 as an internal standard. The chromatographic column was a DB-5ms capillary column, with specifications of 60 m × 0.25 mm × 0.25 μm. The conditions were as follows: injection volume: 1 μL; injection temperature: 260 °C; split ratio: 5:1; carrier gas: helium (99.999%); flow: 1.5 mL/min; column temperature: 140 °C for 5 min, to 180 °C at 10 °C/min, 3 min up to 210 °C at 2 °C/min and up to 280 °C at 5 °C/min, held for 15 min.

### 2.6. Nutrient Digestibility Analysis

For the determination of nutrient digestibility, the bottle contents were passed through previously weighed filter paper, and the residue was washed with hot distilled water. Afterward, the filter paper containing the residue was oven-dried until a constant weight at 105 °C. The crude protein (CP) contents were analyzed according to method 976.05 of the Association of Official Analytical Chemists [22]. Neutral detergent fiber (NDF) and acid detergent fiber (ADF) in the feed substrate and residues were determined according to the methods described by Van Soest et al. [23]. The DM, CP, ADF, and NDF digestibility (%) was calculated as: digestibility (%) = (1 − weight of residue after digestion/weight of substrate before digestion) × 100.

### 2.7. DNA Extraction and the Microbial Population Analysis

The total microbial DNA was extracted from rumen fluid using the cetyltrimethylammonium bromide (CTAB) method as described by Denman and McSweeney [24]. The determination of the number of microorganisms in rumen fluid was carried out using quantitative real-time PCR (qRT-PCR), with a high throughput sequencing instrument [25]. The primers used for total bacteria were UniF(306) (GTGSTGCAYGGYYGTCGTCA) and UniR(309) (ACGTCRTCCMCNCCTTCCTC) [26]; for methanogens, Met630F(501) (GGATTAGATACCCSGGTAGT) and Met803R (GTT-GARTCCAATTAAACCGCA) [18]; for Protozoa, ProtozoaF (GCTTTCGWTGGTAGTGTATT) and PotozoaR (CTTGCCCTCYAATCGTWCT) [27]; for fungi, FungiF (GAGGAAGTAAAAGTCGTAACAAGGTTTC) and FungiR (CAAATTCACAAAGGGTAGGATGATT) [24]. PCR was performed using the SYBRGreen fluorescent dye in a Roche light cycler 480 RT-PCR machine (Roche, Basel, Switzerland). The results were then transformed to log_10_ copies/mL of the sample for further statistical analysis.

### 2.8. 16S rDNA Gene Sequencing and Bioinformatic Analysis

High throughput (Illumina MiSeq) sequencing of the 16S rDNA gene was carried out using barcoded primers for the V3–V4 region (front end primers: 343F TACGGRAGGCAGCAG, backend primers: 798R AGGGTATCTAATCCT). Based on the original data, the paired reads were spliced into a sequence according to the overlapping relationship between raw reads, and then the samples were identified and distinguished according to the barcode tag sequence and primer sequence at the beginning and end of the sequence to obtain data for each sample. Finally, the quality of each sample data and the effect of the merge were filtered by quality control to obtain the effective sequence of each sample. The rumen bacterial composition of samples was determined using species annotation and abundance analysis, and further alpha diversity analysis was conducted to determine the differences among samples. Bioinformatic analysis of the OTU data was conducted using the Oebiotech cloud platform (https://cloud.oebiotech.cn/, accessed on 11 November 2022) provided by Shanghai OE Biotech Co., Ltd. (Shanghai, China) to determine the relative abundance, microbial diversity matrices, and other parameters.

### 2.9. Statistical Analysis

Data were analyzed by analysis of variance using a general linear model in SAS software (version 9.2; SAS Institute, Cary, NC, USA). Duncan’s test was used to identify differences (*p* < 0.05) between the means. The Alpha diversity index was calculated using Mothur software (version v.1.30). The microbial Beta diversity was determined using principal coordinate analysis. PERMANOVA amongst all groups was performed (using 999 permutations). *p*-values less than 0.05 were considered statistically significant.

## 3. Results

### 3.1. In Vitro Ruminal Gas Production

Although no significant (*p* > 0.05) change in gas production (total gas, H_2_, and CH_4_) was observed in response to the treatments (Table 3), the total gas and CH_4_ decreased and H_2_ increased when TSO was added, compared to the control.

### 3.2. In Vitro Rumen Fermentation Parameters

Compared to CT, T2 and T3 had lower (*p* < 0.05) acetate and total VFA but higher MCP (*p* < 0.05) content (Table 4). The lowest NH_3_-N content was found in T1 and T2 (*p* < 0.05), followed by T3 and CT. The content of valerate in T1 was higher (*p* < 0.05) than in the control. There was no effect (*p* > 0.05) of TSO on the pH, propionate, isobutyrate, butyrate, isovalerate, and acetate/propionate in vitro in water buffalo rumen fluid.

### 3.3. In Vitro Ruminal Nutrient Digestibility

Treatments increased (*p* < 0.05) the DM digestibility (Table 5); the highest percentage (56.68%) was observed for T2 followed by T3 (56.26%) and T1 (55.50%), compared to CT. The T2 showed higher CP digestibility than the CT group (*p* < 0.05). However, the results revealed no effect (*p* > 0.05) of TSO on the NDF and ADF digestibility.

### 3.4. Fatty Acid Composition

The effects of TSO supplementation on the fatty acid content of rumen fluid are shown in Table 6. T3 had a higher C18:3n6 concentration than T1 (*p* < 0.05). Supplementation with TSO increased the concentrations of C18:2n6c, C18:2 trans-10, cis-12, and C20:4n6 compared to those in CT, whereas T2 and T3 were significantly higher in CT and T1, respectively (*p* < 0.05). However, compared to CT, TSO supplementation decreased the C18:1n9c concentration, which was significantly lower in T3 (*p* < 0.05). The C18:0 concentration in T2 was significantly lower than that in CT (*p* < 0.05). C20:3n3 and C24:0 concentrations were significantly lower in CT and T1 than in T3 (*p* < 0.05). The C22:5n6 concentrations in T1 and T2 were higher than those in CT and T3, respectively (*p* < 0.05).

### 3.5. Rumen Microbial Populations

The number of total bacteria in T2 was lower (*p* < 0.05) compared to CT (Table 7). However, the results revealed no effect (*p* > 0.05) of TSO on the microbial populations of methanogens, protozoa, and fungi.

### 3.6. Rumen Bacterial Diversity

**Figure 1:**
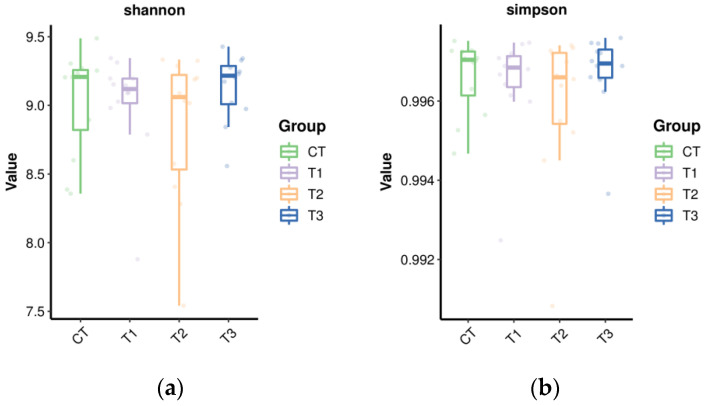

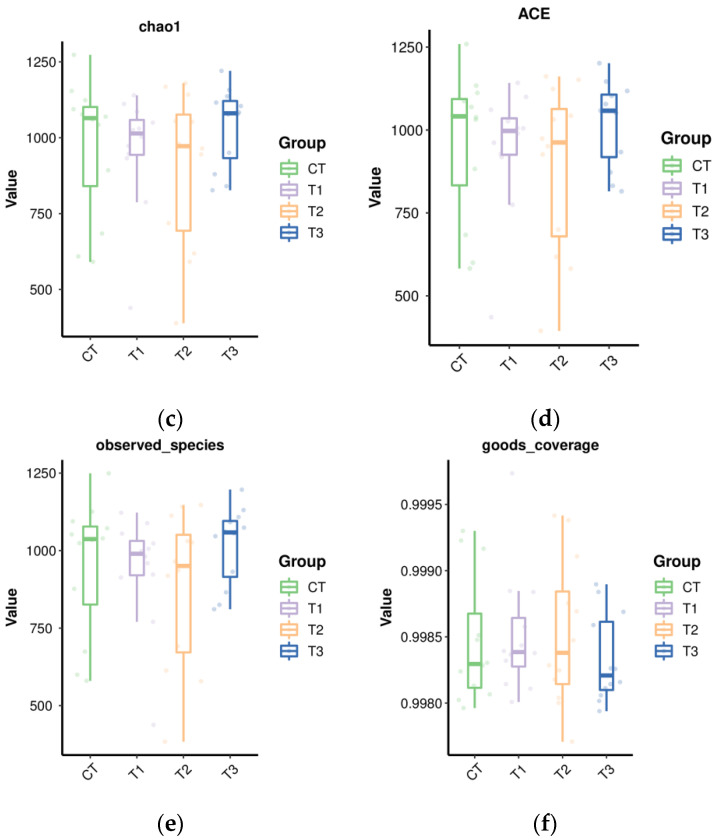
Changes in alpha diversity indices in the bacterial community in the in vitro rumen fermentation with increasing tea seed oil supplementation. (**a**) Shannon index; (**b**) Simpson index; (**c**) Chao index; (**d**) Ace index; (**e**) Observed_species index; (**f**) goods_coverage.

Bacterial alpha diversity parameters in the present study are shown in Figure 1. There were no differences (*p* > 0.05) in the Shannon, Simpson, Chao, Ace, observed_species, and goods_coverage index, indicating that the sequencing depth was desirable for the current analysis and that TSO supplementation did not affect the richness and diversity of the bacterial community.

**Figure 2:**
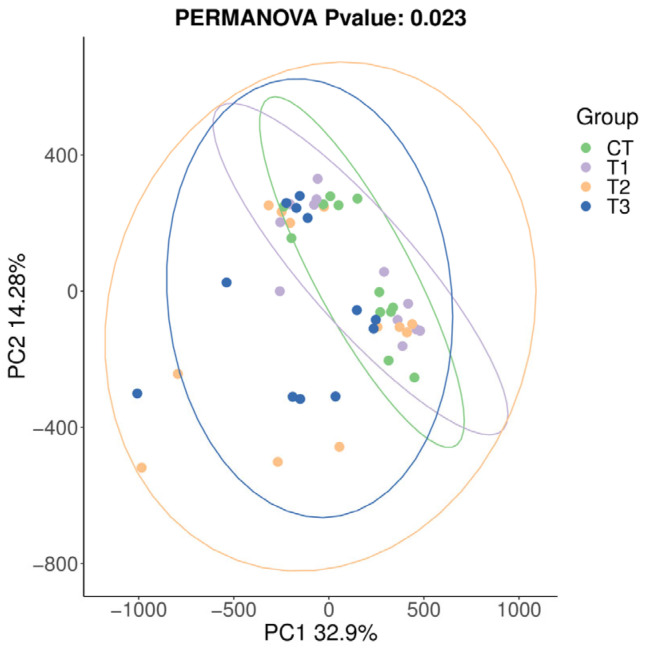
Principal coordinate analysis of the bacterial community in the in vitro rumen fermentation with increasing tea seed oil supplementation.

The composition of the bacterial community was compared between various treatments and analyzed using beta diversity analysis. As illustrated by PCoA (Figure 2), the treatment and control groups did not cluster separately, indicating a similarity in the composition of the rumen flora.

### 3.7. Relative Abundance of Bacterial Populations

**Figure 3:**
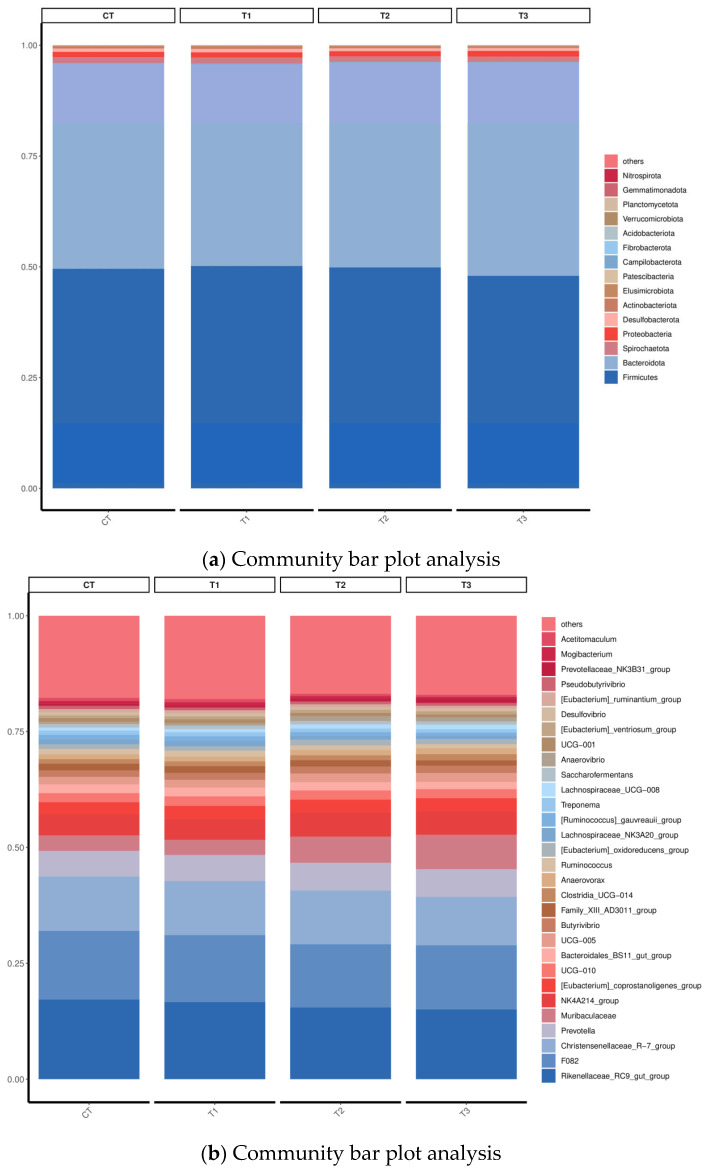
Relative abundance of rumen microflora of buffalo at the phylum level (**a**) and genus level (**b**).

The relative abundance of microorganisms in the rumen contents of water buffalo at the phylum and genus levels is shown in Figure 3. *Firmicutes* and *Bacteroidota* were dominant phyla that accounted for more than 85% of the whole rumen bacteriome (Figure 3a). Other major bacterial phyla were *Spirochaetota*, *Proteobacteria*, *Desulfobacterota*, *Actinobacteriota*, *Elusimicrobiota*, *Patescibacteria*, *Campylobacterota*, *Fibrobacteriota*, *Acidobacteriota*, *Verrucomicrobiota*, *Planctomycetota*, *Gemmatimonadiota* and *Nitrospirota*. However, no difference in the relative abundance of *Firmicutes* and *Bacteroidota* among the groups was observed (*p* > 0.05).

**Figure 4:**
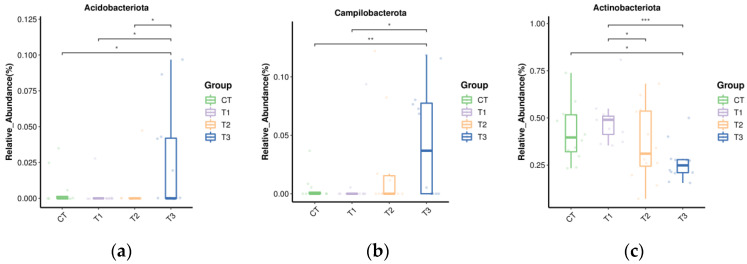
Effect of tea seed oil supplementation on the relative abundance of different bacteria phyla. Asterisk sign was used when the *p* values were less than 0.05 (* 0.01 < *p* ≤ 0.05, ** 0.001 < *p* ≤ 0.01, *** *p* ≤ 0.001). (**a**) Acidobacteriota; (**b**) Campylobacterota; (**c**) Actinobacteriota.

The relative abundance of *Acidobacteriota* in T3 was higher than in other groups (*p* < 0.05) (Figure 4a). Compared with the CT and T1 group, the relative abundance of *Campylobacterota* was higher in T3 (Figure 4b) (*p* < 0.05). The relative abundance of *Actinobacteriota* was lower in T3 than in CT and T1 (*p* < 0.05) (Figure 4c).

**Figure 5:**
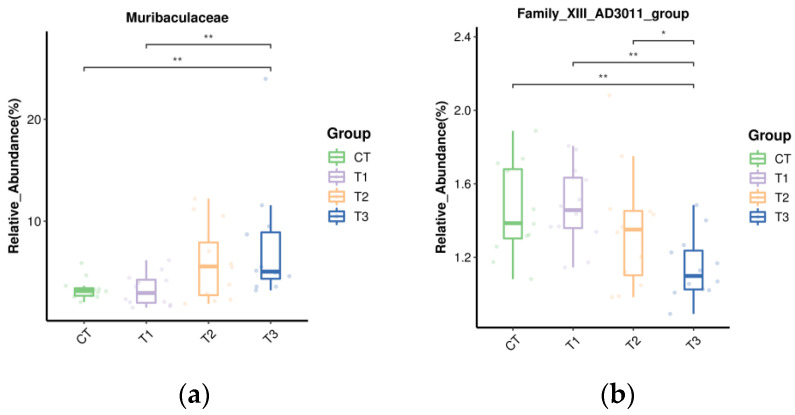

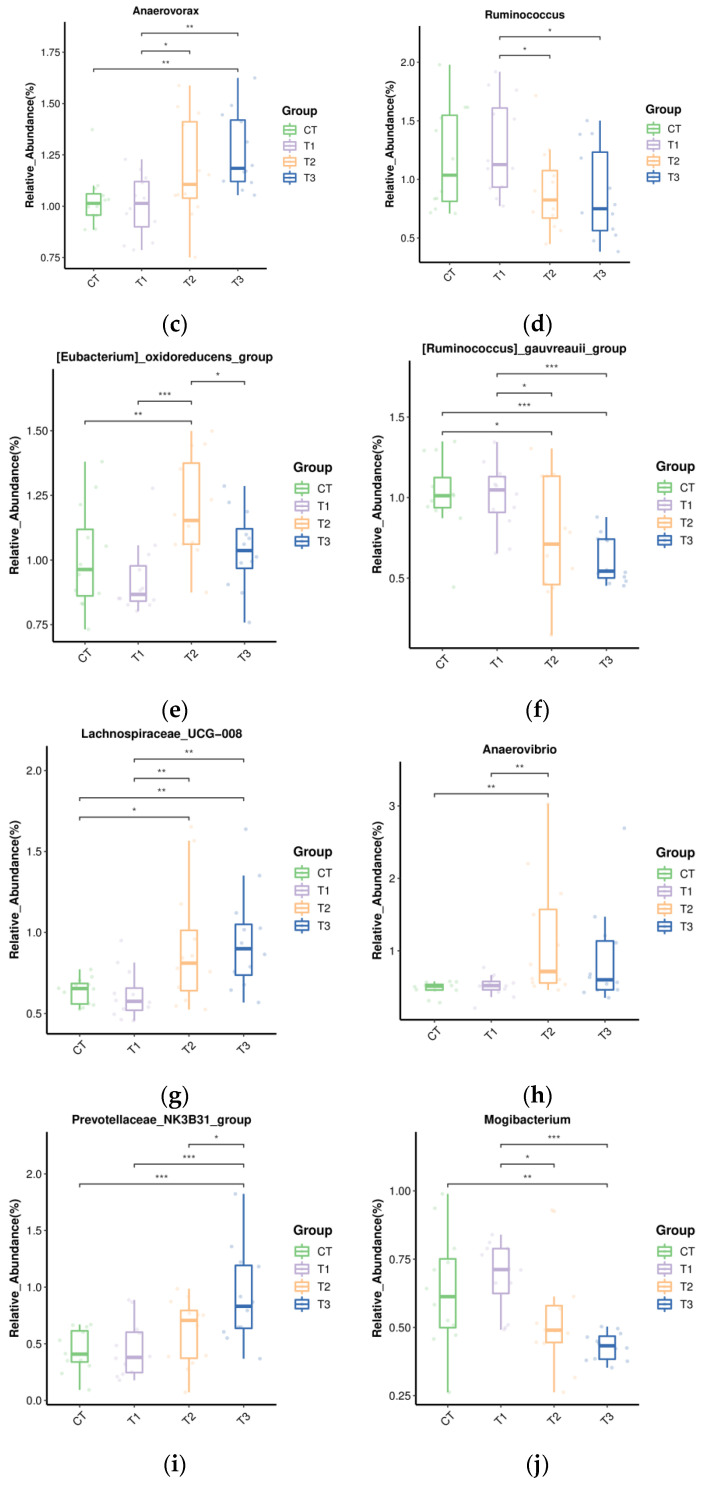
Effect of tea seed oil supplementation on the relative abundance of different bacteria genera. Asterisk sign was used when the p values were less than 0.05 (* 0.01 < *p* ≤ 0.05, ** 0.001 < *p* ≤ 0.01, *** *p* ≤ 0.001). (**a**) Muribaculaceae; (**b**) Family_XIII_AD3011_group; (**c**) Anaerovorax; (**d**) Ruminococcus; (**e**) [Eubacterium]_oxidoreductens_group; (**f**) [Ruminococcus]_gauvreauii_group; (**g**) Lachnospiraceae_UCG-008; (**h**) Anaerovibrio; (**i**) Prevotellaceae_NK3B31_group; (**j**) Mogibacterium.

The relative abundance of major bacterial genera is shown in Figure 3b. *Rikenellaceae_RC9_gut_group* was the dominant genus with the highest relative abundance among the four groups, and the secondary dominant genera were all less than 11%, including *F082*, *Christensenellaceae_R-7_group*, *Prevotella*, *Muribaculaceae*, *NK4A214_group*, [*Eubacterium*]_*oxidoreducens*_*group*, *UCG-011*, and *Bacteroidales_BS11_gut_group*, etc. The relative abundance of Muribaculaceae in T3 was increased compared with CT and T1 groups (*p* < 0.05) (Figure 5a). However, the relative abundance of Family_XIII_AD3011_group in the T3 group was lower than in other groups (Figure 5b) (*p* < 0.05). The relative abundance of *Anaerovorax* was higher in T3 as compared to the CT and T1 group (*p* < 0.05) (Figure 5c), and T2 was higher than T1 (*p* < 0.05). The relative abundance of *Ruminococcus* was higher in T1 than in T2 and T3 (*p* < 0.05) (Figure 5d). The relative abundance of [Eubacterium]_oxidoreductens_group was higher in T2 than in the other groups (*p* < 0.05) (Figure 5e). The CT and T1 groups had higher [*Ruminococcus*]_*gauvreauii*_group relative abundance, but lower relative abundance of Lachnospiraceae_UCG-008 compared to T2 and T3, respectively (*p* < 0.05) (Figure 5f,g). The relative abundance of *Anaerovibrio* in T2 was higher than in CT and T1 (*p* < 0.05) (Figure 5h). The relative abundance of Prevotellaceae_NK3B31_group in the T3 group was higher than in the other groups (Figure 5i) (*p* < 0.05). However, the relative abundance of *Mogibacterium* in T3 was lower than in CT and T1; T2 was lower than T1 (Figure 5j) (*p* < 0.05).

## 4. Discussion

In the current study, SGR had higher DM, NDF, and ADF contents relative to SR, indicating that SGR could be used as a feed resource for ruminants. SR had higher CP (29.32% DM), but lower DM, DM, NDF, and ADF contents. One of the methods facilitating the reuse of residues is their application in animal feed industries. Accordingly, using SGR and SR, by-products of industry and agriculture, as animal feed, not only reduces production costs and is a cost-effective protein supplement feed, but also improves the utilization of feed resource waste and ultimately makes the feed more nutritionally balanced.

Gas production in the rumen is basically the result of the fermentation of carbohydrates into VFA and substantial changes in carbohydrate fractions are reflected by the total gas produced [28,29,30,31]. H_2_ is produced in the rumen during the microbial fermentation of dietary carbohydrates and is consumed as an energy source by H_2_-using microbes, especially the CH_4_-forming methanogens [32]. Ruminal CH_4_ is produced through the CO_2_–H_2_ reduction pathway under the action of methanogens [21]. Natural additives have the potential to modulate rumen fermentation and improve animal performance [33,34].

In the present study, although dietary supplementation with TSO did not markedly alter the gas production during in vitro incubation, total gas production and CH_4_ in the treatment group were lower than in the control group. This might be associated with the potential of bioactive compounds of TSO to act as rumen fermentation modifiers to possibly mitigate enteric gas and CH_4_ production.

Rumen health and internal environmental stability are of great significance to ruminants. The rumen pH is an important factor in the process of anaerobic fermentation, which has an obvious effect on the hydrolysis and acidogenesis of rumen microorganisms [35]. In the current study, the changes in ruminal pH of the substrate with or without three different levels of TSO were maintained within the normal range (5.5 to 7.5) [36], indicating that all diets produced stable rumen fermentation levels, which can be attributed to the GSR and SR diets with TSO supplementation that enhance ruminal fermentation capacity. VFA production by ruminal micro-organisms accounts for 60–70% of the metabolizable energy supply in ruminants [37]. The effect of EOs or any feed additive on rumen fermentation is considered positive when there is increased total volatile fatty acid (TVFA) and propionate production and a decreased acetate-to-propionate ratio [38,39]. Some of the studies in the literature indicate a detrimental effect of higher EO inclusion on in vitro rumen fermentation, including a reduction in TVFA production and a reduction in CH_4_ synthesis [40,41,42,43,44,45]. The decrease in VFA and NH_3_-N production at the higher doses of TSO in the present study might be due to a depressing effect on microbial fermentation [46]. Fermentation end products are very complex and the variability in the response of EOs might be due to the chemical composition of EOs affecting their antimicrobial activities. This is highly variable and depends on many factors, such as plant species, stage of growth, parts of a plant, and extraction method [47]. Rumen microorganisms can degrade crude protein from feed and saliva to produce ammonia, small peptides, and amino acids, and use these degradation products as nitrogen sources to synthesize MCP [48,49,50]. In the rumen, the MCP level usually reflects the growth rate and the population of rumen microbes [49]. The increase in MCP observed in response to TSO treatments in the present study is favorable, particularly in the case of the T2 and T3 groups, suggesting the positive effect of TSO on microbial populations.

The digestibility of feed nutrients is influenced by various factors, such as dietary quality, animal feed intake, feed retention time in the rumen, chyme flow rate, and rumen microbial activity [51]. In the current study, treatments increased the DM digestibility; the highest percentage (56.68%) was observed for T2, followed by T3 (56.26%), and T1 (55.50%). This could be due to oil prolonging the residence time of feed in the digestive tract, thereby improving the digestibility and absorption of feed nutrients. The degradation of rumen microorganisms is the main factor influencing protein digestion and utilization; thus, in the rumen of ruminants, protein is first degraded by protein-decomposing bacteria into amino acids, small peptides, and ammonia, and then converted into microbial proteins through energy conversion in the rumen, which are finally utilized by an animal [52]. In the current study, T2 had higher CP digestibility than the CT group. This may be caused by the ability of TSO to promote the growth of protein-decomposing microorganisms by providing protein-degrading enzymes that aid digestion by degrading proteins with complex structures and high molecular weights [53].

Feed lipids are quickly hydrolyzed and free fatty acids are released under the action of microbial lipase after they enter the rumen; unsaturated fatty acids are quickly hydrogenated into saturated fat fatty acids by microorganisms [54,55]. However, due to the incomplete hydrogenation process, a series of intermediate products such as trans11-C18:1, C18:1, conjugated linoleic acid, and its isomers are generated [56]. Furthermore, reductase, isomerase, and rumen esterase also play an important role in fatty acid metabolism, which further protects polyunsaturated fatty acids from microbial hydrogenation [57]. Supplementation with TSO increased the concentrations of C18:3n6, C18:2n6c, C18:2 trans-10, cis-12, and C20:4n6 compared to the control. These positive effects may be related to the oleic-acid-rich composition and the presence of varying bioactive compounds in TSO [58]. However, compared to CT, supplementation with TSO decreased the concentration of C18:1n9c and C18:0. The inhibition effect of fatty acids on rumen fermentation may be due to fatty acids adhering to the feed surface, hindering microorganism decomposition of the feed substrate [59].

The microbial community of the rumen is complex and consists of bacteria, protozoa, archaea, and fungi. It has been established that the composition and activity of the rumen microbiota have a profound impact on the performance, health, and immune system of the host [60,61,62]. Quantitative PCR indicated that TSO treatments did not substantially alter the populations of methanogens, protozoa, and fungi. However, total bacterial populations were markedly decreased when diets were supplemented with TSO at 1% DM. The inhibitory activity of TSO against bacteria has been evidenced by the ability of TSO to form complexes with the cell wall and membrane of bacteria, causing morphological changes in the cell wall and the secretion of extracellular enzymes [63].

Microorganisms in the rumen are important intermediaries such that ruminants can digest nutrients in their diets [64]. The alpha diversity analysis of buffalo rumen flora in this study showed that the coverage of each group was higher than 98%, indicating that the sequencing results truly reflected the species and structural diversity of the rumen bacterial community. However, there were no marked differences in Shannon, Simpson, Chao, Ace, Observed_species, and goods_coverage indices, indicating that the sequencing depth was desirable for the analysis and that TSO supplementation did not affect the richness and diversity of the bacterial community. Beta diversity produced similar clusters in the treatment and control groups, indicating a similarity in rumen flora composition. Ruminant animals have a very diverse bacterial community [65]. In the current study, *Firmicutes* and *Bacteroidota* were the prevalent phyla, which concurs with the results of previous studies [18,21]. The relative abundance of Acidobacteriota, Muribaculaceae, Campylobacterota, *Anaerovorax*, *Ruminococcus*, [*Eubacterium*]_oxidoreducens_group, *Anaerovibrio*, and *Prevotellaceae_NK3B31*_group were increased but *Actinobacteriota*, Family_XIII_AD3011_group, *Lachnospiraceae_UCG-008*, and *Mogibacterium* decreased with the addition of TSO and may indicate an antibacterial potency of TSO [66,67]. The results indicated that the addition of TSO affected the composition of rumen bacteria to a certain extent.

## 5. Conclusions

Our study demonstrated that TSO could regulate the number and diversity of rumen microorganisms through antimicrobial activity, thereby affecting rumen fermentation patterns, reducing methane production, and improving nutrient digestibility. The modulation of fermentation by TSO inclusion altered many ruminal bacterial genera that are associated with feed digestibility and ruminal fermentation characteristics. Thus, an optimal feeding rate for future animal studies appears to be 1% TSO of dietary dry matter for ruminants. However, further in vivo experimental studies should be carried out to determine the optimal dose of TSO in diets for various dietary types and growth stages of ruminants, to evaluate its effect on the adaptive characteristics of rumen microbiota and animal production.

## Figures and Tables

**Table 1:** Chemical composition of *Siraitia grosvenorii* residue and soybean residue.

Items	DM (%)	CP	NDF	ADF	Ash	GE (MJ/kg DM)
		(% DM)				
*Siraitia grosvenorii* residue	89.55	10.76	70.99	54.08	3.30	21.14
Soybean residue	18.48	29.32	25.30	13.74	3.87	19.45

ADF, acid detergent fiber; CP, crude protein; DM, dry matter; GE, gross energy; NDF, neutral detergent fiber.

**Table 2:** Experimental design.

Treatments	Substrate Compositions (%) (Dry Matter Basis)	Tea Seed Oil (%) (Dry Matter Basis)
CT	70% SGR + 30% SR	0
T1	70% SGR + 30% SR	0.5
T2	70% SGR + 30% SR	1
T3	70% SGR + 30% SR	2

**Table 3:** In vitro ruminal gas production of substrates supplemented with tea seed oil.

Item	Groups ^1^				SEM	*p*-Value
	CT	T1	T2	T3		
Gas production (mL/g DM)						
Total gas	87.60	83.49	80.93	81.35	6.2091	0.9351
H_2_	0.17	0.25	0.28	0.27	0.1569	0.9442
CH_4_	21.96	21.56	21.44	21.13	5.5179	0.9996

Means within a row with unlike superscripts differ (*p* < 0.05). ^1^ Groups CT, T1, T2, and T3 were substrates supplemented with 0%, 0.5%, 1%, and 2% of TSO based on dry matter weight, respectively. SEM, standard error of the mean.

**Table 4:** Effects of tea seed oil supplementation on in vitro fermentation characteristics.

Item	Groups ^1^				SEM	*p*-Value
	CT	T1	T2	T3		
pH	6.77	6.80	6.83	7.07	0.1339	0.4248
VFA (mmol/L)						
Acetate	17.64 ^a^	15.96 ^ab^	13.20 ^b^	13.22 ^b^	1.3108	0.0145
Propionate	9.18	9.21	7.63	7.70	0.5320	0.066
Isobutyrate	0.40	0.43	0.34	0.37	0.0409	0.5032
Butyrate	5.35	5.38	4.41	4.44	0.3334	0.0764
Isovalerate	1.14	1.23	1.04	1.04	0.0700	0.1709
Valerate	0.77 ^b^	0.91 ^a^	0.81 ^ab^	0.81 ^ab^	0.0408	0.0259
Total	34.44 ^a^	33.13 ^ab^	27.43 ^b^	27.58 ^b^	2.1680	0.0297
Acetate/propionate	1.89	1.72	1.75	1.68	0.0718	0.2033
NH_3_-N (mg/100 mL)	14.51 ^a^	12.63 ^b^	12.37 ^b^	13.81 ^a^	0.3494	0.0002
MCP (mg/mL)	3.82 ^b^	3.94 ^ab^	4.33 ^a^	4.32 ^a^	0.1443	0.0274

Means within the same row with superscripts differ (*p* < 0.05). ^1^ Groups CT, T1, T2, and T3 were substrates supplemented with 0%, 0.5%, 1%, and 2% of TSO based on dry matter weight, respectively. VFA, volatile fatty acid; NH3-N, ammonia nitrogen; MCP, microbial crude protein; SEM, standard error of the mean.

**Table 5:** Effects of substrates supplemented with tea seed oil on in vitro ruminal nutrient digestibility.

Item	Groups ^1^				SEM	*p*-Value
	CT	T1	T2	T3		
Nutrient digestibility (%)						
DM	52.48 ^b^	55.50 ^a^	56.68 ^a^	56.26 ^a^	0.9716	0.0163
CP	61.86 ^b^	62.32 ^ab^	64.87 ^a^	64.03 ^ab^	0.5039	0.0371
NDF	42.63	44.34	46.92	43.46	0.7670	0.1748
ADF	35.15	37.22	39.02	36.94	0.8060	0.6229

Means within a row with unlike superscripts differ (*p* < 0.05). ^1^ Groups CT, T1, T2, and T3 were substrates supplemented with 0%, 0.5%, 1%, and 2% of TSO based on dry matter weight, respectively. ADF, acid detergent fiber; CP, crude protein; DM, dry matter; NDF, neutral detergent fiber. SEM, standard error of the mean.

**Table 6:** Effects of tea seed oil on in vitro rumen fatty acid profile of water buffalo (μg/mL).

Items	Groups ^1^				SEM	*p*-Value
	CT	T1	T2	T3		
C12:0	1.22	1.03	1.26	1.06	0.1555	0.6528
C13:0	0.11	0.09	0.14	0.16	0.4366	0.7274
C14:1n5	0.16	0.15	0.13	0.13	0.0193	0.6079
C14:0	3.57	3.29	4.03	3.43	0.3280	0.4225
C15:1n5	0.26	0.29	0.25	0.35	0.0318	0.4152
C15:0	0.32	0.32	0.35	0.31	0.0139	0.6058
C16:0	73.66	66.62	81.90	86.63	9.3773	0.4523
C16:1n7	0.27	0.26	0.31	0.29	0.0158	0.1822
C17:0	2.22	2.19	2.59	2.44	0.2625	0.6717
C17:1n7	2.58	2.38	3.11	3.14	0.2723	0.1417
C18:3n6	2.62 ^ab^	2.10 ^b^	3.03 ^ab^	3.20 ^a^	0.3490	0.0353
C18:2n6c	7.72 ^b^	11.41 ^b^	20.46 ^a^	23.28 ^a^	2.0078	<0.0001
C18:2 cis-9,trans-11	7.46	8.34	9.72	9.95	1.2364	0.4790
C18:1n9c	121.88 ^a^	82.84 ^ab^	84.68 ^ab^	58.07 ^b^	15.2327	0.0395
C18:2 trans-10, cis-12	0.60 ^b^	0.64 ^b^	1.30 ^a^	1.53 ^a^	0.2288	0.0107
C18:1n9t	0.68	0.72	0.67	0.52	0.1447	0.7833
C18:0	3.49 ^a^	1.94 ^ab^	1.52 ^b^	2.23 ^ab^	0.5981	0.0270
C20:4n6	0.46 ^b^	0.44 ^b^	0.77 ^a^	0.78 ^a^	0.0728	0.0009
C20:5n3	0.04	0.05	0.10	0.11	0.0328	0.4010
C20:3n3	0.06 ^b^	0.08 ^b^	0.17 ^ab^	0.22 ^a^	0.0374	0.0127
C20:1	3.22	3.54	4.62	4.44	0.4969	0.1655
C20:2n	0.02	0.02	0.03	0.06	0.0142	0.1855
C20:0	0.12	0.12	0.15	0.20	0.0275	0.1343
C21:0	0.20	0.20	0.17	0.18	0.0411	0.9467
C22:6n3	0.06	0.09	0.11	0.20	0.0457	0.1677
C22:5n3	0.48	0.46	0.64	0.90	0.1608	0.1968
C22:5n6	0.08 ^b^	0.20 ^a^	0.31 ^a^	0.05 ^b^	0.0411	0.0002
C22:1n9	1.18	1.60	1.55	1.70	0.1520	0.1144
C22:2n6	0.02	0.02	0.02	0.04	0.0090	0.1119
C22:0	0.14	0.11	0.17	0.21	0.0277	0.1720
C23:0	0.02	0.02	0.03	0.04	0.0080	0.2751
C24:1n9	0.02	0.02	0.01	0.02	0.0057	0.3908
C24:0	0.41 ^b^	0.40 ^b^	0.59 ^ab^	0.74 ^a^	0.0989	0.0487

Means within the same row with unlike superscripts differ (*p* < 0.05). ^1^ Groups CT, T1, T2, and T3 were substrates supplemented with 0%, 0.5%, 1%, and 2% of TSO based on dry matter weight, respectively. SEM, standard error of the mean.

**Table 7:** Effects of tea seed oil supplementation on microbial populations (log10 copies per g of rumen contents).

Items	Groups ^1^				SEM	*p*-Value
	CT	T1	T2	T3		
Total Bacteria	12.82 ^a^	12.62 ^b^	12.79 ^ab^	12.77 ^ab^	0.0620	0.0238
Methanogens	9.67	9.55	9.56	9.65	0.0887	0.7138
Protozoa	8.54	9.54	7.60	8.16	0.0839	0.8298
Fungi	9.13	9.31	9.31	9.19	0.0966	0.5280

Means within the same row with unlike superscripts differ (*p* < 0.05). ^1^ Groups CT, T1, T2, and T3 were substrates supplemented with 0%, 0.5%, 1%, and 2% of TSO based on dry matter weight, respectively. SEM, standard error of the mean.

## Data Availability

Data not available online are available from the authors upon request.
